# Pharmacist-Led Intervention to Enhance Medication Adherence in Patients With Acute Coronary Syndrome in Vietnam: A Randomized Controlled Trial

**DOI:** 10.3389/fphar.2018.00656

**Published:** 2018-06-21

**Authors:** Thang Nguyen, Thao H. Nguyen, Phu T. Nguyen, Ha T. Tran, Ngoc V. Nguyen, Hoa Q. Nguyen, Ban N. Ha, Tam T. Pham, Katja Taxis

**Affiliations:** ^1^Department of Pharmacology and Clinical Pharmacy, Can Tho University of Medicine and Pharmacy, Can Tho, Vietnam; ^2^Department of Clinical Pharmacy, University of Medicine and Pharmacy, Ho Chi Minh City, Vietnam; ^3^Heart Institute of Ho Chi Minh City, Ho Chi Minh City, Vietnam; ^4^Faculty of Public Health, Can Tho University of Medicine and Pharmacy, Can Tho, Vietnam; ^5^Groningen Research Institute of Pharmacy, University of Groningen, Groningen, Netherlands

**Keywords:** acute coronary syndrome, medication adherence, pharmacist-led intervention, randomized controlled trial, Vietnam

## Abstract

**Background:** Patient adherence to cardioprotective medications improves outcomes of acute coronary syndrome (ACS), but few adherence-enhancing interventions have been tested in low-income and middle-income countries.

**Objectives:** We aimed to assess whether a pharmacist-led intervention enhances medication adherence in patients with ACS and reduces mortality and hospital readmission.

**Methods:** We conducted a randomized controlled trial in Vietnam. Patients with ACS were recruited, randomized to the intervention or usual care prior to discharge, and followed 3 months after discharge. Intervention patients received educational and behavioral interventions by a pharmacist. Primary outcome was the proportion of adherent patients 1 month after discharge. Adherence was a combined measure of self-reported adherence (the 8–item Morisky Medication Adherence Scale) and obtaining repeat prescriptions on time. Secondary outcomes were (1) the proportion of patients adherent to medication; (2) rates of mortality and hospital readmission; and (3) change in quality of life from baseline assessed with the European Quality of Life Questionnaire – 5 Dimensions – 3 Levels at 3 months after discharge. Logistic regression was used to analyze data. Registration: ClinicalTrials.gov (NCT02787941).

**Results:** Overall, 166 patients (87 control, 79 intervention) were included (mean age 61.2 years, 73% male). In the analysis excluding patients from the intervention group who did not receive the intervention and excluding all patients who withdrew, were lost to follow-up, died or were readmitted to hospital, a greater proportion of patients were adherent in the intervention compared with the control at 1 month (90.0% vs. 76.5%; adjusted OR = 2.77; 95% CI, 1.01–7.62) and at 3 months after discharge (90.2% vs. 77.0%; adjusted OR = 3.68; 95% CI, 1.14–11.88). There was no significant difference in median change of EQ-5D-3L index values between intervention and control [0.000 (0.000; 0.275) vs. 0.234 (0.000; 0.379); *p* = 0.081]. Rates of mortality, readmission, or both were 0.8, 10.3, or 11.1%, respectively; with no significant differences between the 2 groups.

**Conclusion:** Pharmacist-led interventions increased patient adherence to medication regimens by over 13% in the first 3 months after ACS hospital discharge, but not quality of life, mortality and readmission. These results are promising but should be tested in other settings prior to broader dissemination.

## Introduction

In patients with ischemic heart diseases (IHDs), medication adherence improves health outcomes and reduces costs (Horne et al., 2005; Simpson et al., 2006; Bitton et al., 2013; Chowdhury et al., 2013). IHDs are the world’s biggest killer, accounting for 8.9 million deaths in 2015 (GBD 2015 DALYs and HALE Collaborators, 2016; World Health Organization, 2017b). Over 80% of those occur in low-income and middle-income countries (LMICs), such as Vietnam (Dugani and Gaziano, 2016). IHDs comprise stable angina and acute coronary syndrome (ACS) which is the dominant cause of IHD deaths (Moran et al., 2014). Survivors of ACS have an increased risk of recurrent infarctions and their annual death rate is up to six times higher than in healthy people of the same age (World Health Organization, 2017a). A substantial proportion of people are non-adherent to cardiovascular medications (Chowdhury et al., 2013), ranging from 14 to 46% in patients discharged from the hospital after an ACS, higher rates are especially reported for LMICs (Bowry et al., 2011; Akeroyd et al., 2015).

Many types of interventions to improve medication adherence in patients with IHDs have been developed. Of these, a substantial proportion was driven by pharmacists (Santo et al., 2016). In addition to medication dispensing, pharmacists can provide medication education and disease management for patients (Kang et al., 2016). Systematic reviews on the effect of pharmacists (Cai et al., 2013; Kang et al., 2016) have shown a positive impact on outcomes in patients with IHDs. In Vietnam, the extended clinical role of pharmacists in improving the medicine use process has been recognized (Nguyen et al., 2014). However, there is a lack of research on pharmacist-led interventions in ACS which is one of the leading causes of deaths in Vietnam (Vietnam Ministry of Health, 2013). Therefore, we aimed to assess whether a pharmacist-led multifaceted intervention enhances medication adherence in patients with ACS and reduces mortality and hospital readmission.

## Materials and Methods

### Study Design Overview

We conducted a randomized, controlled trial with concealment of allocation and blinded outcome assessment at the Heart Institute of Ho Chi Minh City in Vietnam. The study was approved by the institutional biomedical research ethics committee and was registered at ClinicalTrials.gov (NCT02787941). Written informed consent was obtained from each study participant.

### Study Setting, Population, and Recruitment

The Heart Institute of Ho Chi Minh City was created in 1992 with the mission to offer high-quality care to Vietnamese patients suffering from heart diseases. Since 1992, over 25,000 patients with complicated heart problems have been treated. In general, the heart institute nowadays performs heart surgeries to the same level as done in high-income countries. Furthermore, the institute runs cooperation programs to transfer technology and heart surgery techniques to provincial hospitals (Tuoi Tre News, 2015).

At the study hospital usual care for patients with ACS was as follows. After discharge, patients with ACS were followed up at a public or private health care center as an outpatient. Appointments were scheduled every two to four weeks to assess health status and progress of the disease, issue a new prescription for medication, and schedule the next appointment. The patient had their medication dispensed at the hospital pharmacy free of charge (if they have a social health insurance) or at any private pharmacy with payment. Social health insurance in Vietnam is the national insurance coverage paid by the Vietnamese government for medical and surgical expenses incurred by the insured. The contributions are paid by the government completely or partially. Prescriptions might be redeemed up until the date of the next appointment. This is the normal process of care for a patient after an ACS in Vietnam.

Recruitment was between 1st November 2015 and 31st October 2016 in Ho Chi Minh City. The follow-up ended on 31st January 2017. Patients admitted with ACS as the primary reason for hospital admission were screened for eligibility. We included patients who survived during hospitalization with one of the following diagnoses according to the coding of the International Classification of Diseases, 10th revision (ICD-10): unstable angina (I20.0), acute myocardial infarction (I21) or subsequent myocardial infarction (I22) (World Health Organization, 2016). We excluded patients who (1) participated already in another medication adherence study; (2) were discharged without a prescription; (3) had considerable cognitive impairment; (4) were unable to communicate in Vietnamese; (5) were unable to identify their own medications; (6) could not provide a telephone number; or (7) stayed in the hospital three days or less (because this period was too short for recruitment, baseline data collection, and intervention).

### Randomization and Intervention

Randomization was performed in advance using an online random number generator (randomization.com). Eligible patients were stratified by age (<65 and 65 years or higher) and sex (male and female), and were randomized into two parallel groups in a 1:1 ratio via block technique with random permuted blocks of 2, 4, or 6 patients. The control group received usual care. The intervention consisted of a pharmacist-delivered multifaceted intervention in addition to usual care. Investigators who performed patient recruitment had been concealed the sequence until the intervention was assigned. Outcome assessors were blinded; patients and pharmacists performing interventions could not be blinded due to the nature of the intervention.

The multifaceted intervention comprised two counseling sessions. At the first counseling, a pharmacist performed a 30-min in-person counseling within 1 week before discharge including: (1) assessment and giving advice on basic knowledge of ACS: definition, risk factors, possible cardiac events, and prevention; (2) assessment of past experiences of using medications, encouragement and tailored advice; (3) providing medication aids including pill organizer and drug information leaflet; (4) teaching back and correcting misunderstanding (Appendix 1). At the second counseling, the pharmacist performed a 30-min telephone counseling within 2 weeks after discharge including: (1) assessment of general and medication-related issues patients concerning; (2) encouragement and tailored advice; (3) teaching back and correcting misunderstanding (Appendix 2).

### Data Collection

The process of data collection and management at baseline and during the follow-up period was summarized in Appendix 3. We collected data using the instruments as follows: the eight-item Morisky Medication Adherence Scale (MMAS-8), the European Quality of Life Questionnaire – 5 Dimensions – 3 Levels (EQ-5D-3L), the Beliefs about Medicines Questionnaire – Specific (BMQ-S), the Mini–Mental State Examination (MMSE), and three predefined data collection forms. The MMAS-8 is an 8-item questionnaire designed to facilitate identification of barriers to and behaviors associated with adherence to medication. Response choices are yes/no for items 1 through 7, and a 5-point Likert response scale for the last item (Morisky et al., 2008). The EQ-5D-3L comprises the following 5 dimensions: mobility, self-care, usual activities, pain/discomfort, and anxiety/depression. Each dimension has 3 levels: no problem, some problems, and extreme problems (EuroQol Group, 2015). The BMQ Specific assesses patients’ beliefs about the particular medications prescribed for them, comprising two subscales: Specific Necessity and Specific Concerns. Each item of the BMQ subscales is scored on a 5-point Likert scale ranging from 1 (strongly disagree) to 5 (strongly agree) (Horne et al., 1999). The MMSE is a 30-point scale that assesses several domains of cognition including memory, orientation, and arithmetic. A lower score indicates greater cognitive impairment (Folstein et al., 1975; Leggett et al., 2013). Data collection form 1 included patients and their caregivers’ contact information, education level, marital status, and ability to identify medications. Data collection form 2 included patients’ baseline characteristics: demographic characteristics, coronary artery disease risk factors, medical history and comorbidities, discharge diagnoses, in-hospital invasive procedures, and discharge medications. Data collection form 3 included the information of complying with medical visits and clinical adverse events.

### Study Outcomes

The primary outcome was predefined in the protocol as the proportion of patients who adhered to cardioprotective medications at 1 month after discharge. We defined patient adherence to cardioprotective medications as returning for their scheduled outpatient appointments (complying with medical visits) and having an MMAS-8 score of six or higher at 1-month follow-up. Complying with medical visits was determined based on patient reports and medical records. Patients being two days or later for their outpatient appointment were considered as non-compliant. We included this as a measure of patient adherence as patients had to return for their appointments to obtain their follow-up prescriptions on time.

The secondary outcomes were (1) the proportion of patients who adhered to cardioprotective medications at 3 months after discharge; (2) the proportion of patients readmitted to hospital of any cause at 3 months; (3) the proportion of patients dying of any cause at 3 months; and (4) change in quality of life from baseline assessed with EQ-5D-3L at 3 months.

### Analysis

For sample size calculations, we assumed 70% adherence (Nguyen et al., 2016), alpha = 0.05, and beta = 0.20. Prior pharmacist-led interventions had achieved absolute improvements in adherence of 15% to 35% (Ho et al., 2014; Lourenco et al., 2014; Oliveira-Filho et al., 2014). In order to detect a 20% difference in adherence between the control and intervention groups and to account for 20% loss to follow-up, we enrolled at least 152 patients (76 per group).

We performed two analyses. **Analysis 1** included all patients excluding patients from the intervention group who did not receive the intervention and excluding all patients who withdrew, were lost to follow-up, died or were readmitted to hospital. **Analysis 2** included all patients who survived and were not readmitted to hospital using multiple imputations to impute missing outcomes of patients who discontinued and were lost to follow-up. Regression models with five iterations were performed to produce five versions of the dataset, each containing its own set of imputed values. The parameter estimates for all of the imputed datasets were pooled, providing estimates that are generally more accurate than they would be with only one imputation.

Data were presented as absolute numbers, percentages, means with standard deviations (SDs), or medians with interquartile ranges (IQRs) as appropriate. Characteristics, quality of life, and mortality/readmission to hospital of patients randomized to the intervention and control groups were compared using Chi-square test or Fisher’s exact test for all categorical variables and Independent *t*-test or Mann–Whitney test for all continuous variables. Univariable and multivariable logistic regression models were used to estimate the odds ratio (OR) with 95% confidence interval (CI) of the intervention for adherence outcomes. The change in quality of life which was assessed using McNemar test (categorical) and Wilcoxon signed rank test (continuous). All tests were two-sided. *P*-values of 0.05 or less were considered statistically significant. Analyses were performed using the Statistical Package for the Social Sciences, version 20th (SPSS 20).

## Results

**FIGURE 1:**
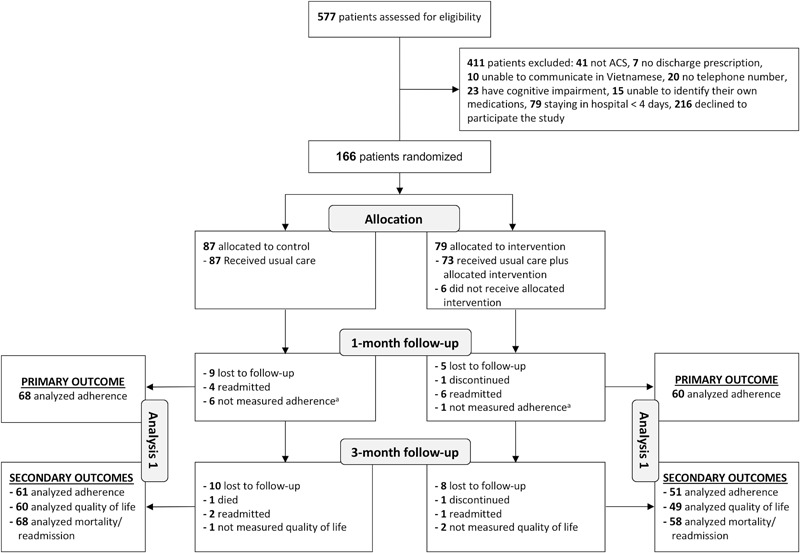
Flowchart of the study population. ACS, acute coronary syndrome; ^a^Patients who were only excluded from the analysis of adherence 1 month after discharge.

Of 577 patients assessed for eligibility, 166 (28.8%) patients were included; 195 (33.8%) patients were excluded due to exclusion criteria; and 216 (37.4%) declined to participate. Of 166 patients included, 87 were randomized to the control condition and 79 to the intervention condition. A total of 153 (92.2%) and 126 (75.9%) completed the 1-month and 3-month follow-ups, respectively. Six patients in the intervention group did not receive the intervention due to severe illness. Two patients who discontinued to participate refused to provide the reason. Reasons for loss to follow-up of 32 patients were not available because we could not contact patients or their relatives (**Figure 1**).

The mean age (SD) was 61.2 (9.6) years, 72.3% were males, and 84.3% had social health insurance. The majority of patients had a discharge diagnosis of non-ST-segment elevation ACS (75.3%) and more than two comorbidities (53.6%). During the hospitalization, median (IQR) of the EQ-5D-3L index value was 0.726 (0.573; 1) and 54.2% patients underwent percutaneous coronary intervention (PCI). No differences in the characteristics of demographics, coronary artery disease (CAD) risk factors, medical history, comorbidities, type of ACS, in-hospital revascularization, discharge medications, BMQ Specific scoring, and EQ-5D-3L scoring were found among groups (**Table 1**).

**Table 1:** Baseline characteristics of the study population.

			Group			
Patient characteristic	Overall (*N* = 166) n (%)		Control (*N* = 87) n (%)		Intervention (*N* = 79) n (%)		*p-value^a^*
***Demographics and general characteristics***							
Age, mean (SD)	61.2	(9.6)	59.8	(8.8)	62	(8.4)	0.221^b^
Ages ≥65	57	(34.3)	28	(32.2)	29	(36.7)	0.540
Male	120	(72.3)	59	(67.8)	61	(77.2)	0.177
Social health insurance	140	(84.3)	73	(83.9)	67	(84.8)	0.873
Education grade ≥6	132	(79.5)	66	(75.9)	66	(83.5)	0.221
Married	150	(90.4)	75	(86.2)	75	(94.5)	0.057
***CAD risk factors***							
CAD family history	3	(1.8)	2	(2.3)	1	(1.3)	1.000^d^
Hypertension	121	(72.9)	58	(66.7)	63	(79.7)	0.058
Diabetes	45	(27.1)	22	(25.3)	23	(29.1)	0.580
Dyslipidemia	41	(24.7)	20	(23.0)	21	(26.6)	0.592
Smoking	71	(43.0)	34	(39.5)	37	(46.8)	0.344
***Medical history and comorbidities***							
Prior MI/stroke	22	(13.3)	11	(12.6)	11	(13.9)	0.808
Prior PCI/CABG	29	(17.5)	16	(18.4)	13	(16.5)	0.743
Heart failure	4	(2.4)	3	(3.4)	1	(1.3)	0.622^d^
Renal failure	15	(9.0)	7	(8.0)	8	(10.1)	0.641
Peptic ulcer	50	(30.1)	26	(29.9)	24	(30.4)	0.945
Asthma/COPD	6	(3.6)	3	(3.4)	3	(3.8)	1.000^d^
Comorbidities ≥2	89	(53.6)	42	(48.3)	47	(59.5)	0.148
***Type of ACS***							
NSTEACS	125	(75.3)	63	(72.4)	62	(78.5)	0.365
STEACS	41	(24.7)	24	(27.6)	17	(21.5)	
***In-hospital revascularization and discharge medications***							
PCI/CABG	90	(54.2)	45	(51.7)	45	(57.0)	0.499
Antiplatelet agent	163	(98.2)	84	(96.6)	79	(100)	0.247^d^
Beta blocker	114	(68.7)	62	(71.3)	52	(65.8)	0.450
ACEI/ARB	158	(95.2)	83	(95.4)	75	(94.9)	1.000^d^
Statin	161	(97.0)	85	(97.7)	76	(96.2)	0.670^d^
Number of medications, mean (SD)	8.0	(1.9)	7.9	(2.0)	8.1	(1.7)	0.533^b^
***BMQ Specific scoring***							
BMQ Specific Necessity, median (IQR)	23	(21; 25)	21	(19; 23)	20	(14; 24)	0.936^c^
BMQ Specific Concern, median (IQR)	11	(9; 14)	10	(9; 13)	12	(9; 15)	0.292^c^
***EQ-5D-3L scoring***							
Mobility^e^	52	(31.3)	33	(37.9)	19	(24.1)	0.054
Self-care^e^	30	(18.1)	15	(17.2)	15	(19.0)	0.770
Usual activities^e^	33	(19.9)	18	(20.7)	15	(19.0)	0.784
Pain/ discomfort^e^	62	(37.3)	35	(40.2)	27	(34.2)	0.421
Anxiety/ depression^e^	65	(39.2)	33	(37.9)	32	(40.5)	0.734
EQ-5D-3L index value, median (IQR)	0.726	(0.573; 1)	0.694	(0.549; 1)	0.766	(0.605; 1)	0.111^c^

*ACEI/ARB, angiotensin-converting enzyme inhibitors or angiotensin II receptor blockers; BMQ, the Beliefs about Medicines Questionnaire; CAD, coronary artery disease; COPD, chronic obstructive pulmonary disease; EQ-5D-3L, the European Quality of Life Questionnaire – 5 Dimensions – 3 Levels; IQR, interquartile range; NSTEACS, non-ST elevation acute coronary syndrome; PCI/CABG, percutaneous coronary intervention/coronary artery bypass grafting; STEACS, ST evaluation acute coronary syndrome. ^*a*^Using Chi-square test if other tests were not mentioned. ^*b*^Using Independent *t*-test. ^*c*^Using Mann–Whitney test. ^*d*^Using Fisher’s exact test. ^*e*^Patients reported having problems with each dimension of EQ-5D-3L.*

In **Analysis 1**, a greater proportion of patients were adherent in the intervention group compared with the control group at 1 month (90.0% vs. 76.5%; adjusted OR = 2.77; 95% CI, 1.01–7.62) and at 3 months after discharge (90.2% vs. 77.0%; adjusted OR = 3.68; 95% CI, 1.14–11.88) (**Tables 2, 3**). There was no covariate associated with adherence at 1 month; but at 3 months patients who were male (adjusted OR = 0.16; 95% CI, 0.03–0.83) or who had more concerns about medicines (higher scores of BMQ Specific Concern) (adjusted OR = 0.89; 95% CI, 0.79–0.99) were less likely to be adherent. Description of non-adherence was presented in Appendix 4.

**Table 2:** Patient adherence at the 1st month after discharge.

	Control n (%)		Intervention n (%)		Absolute difference in proportions, % (95% CI)		Univariable analysis			Multivariable analysis^b^		
							OR	95% CI	*p*	OR	95% CI	*p*
***Analysis 1^a^ (N = 68 control and 60 intervention patients)***								
Adherence	52	(76.5)	54	(90.0)	13.5	(7; 26.4)	2.77	1.01–7.62	**0.043**	2.77	1.01–7.62	**0.049**
Non-adherence	16	(23.5)	6	(10.0)								
***Analysis 2 (N = 83 control and 71 intervention patients)***								
Adherence	57	(68.7)	59	(83.1)	15	(1.6; 28.4)	2.32	1.00–5.36	**0.049**	2.35	1.00–5.52	**0.050**
Non-adherence	26	(31.3)	12	(16.9)								

*Abbreviations: CI, confidence interval; OR, odds ratio. ^*a*^Sample of Analysis 1 excluding patients who were not measured adherence at 1 month. ^*b*^Using multivariable backward stepwise logistic regression models. Variables entered at the first step: intervention status, age, gender, health insurance, education grade ≥6, marital status, number of comorbidities ≥2, type of ACS, BMQ Specific Necessity and Concern scores, and PCI/CABG.*

**Table 3:** Patient adherence at the first 3 months after discharge.

	Control n (%)		Intervention n (%)		Absolute difference in proportions, % (95% CI)		Univariable analysis			Multivariable analysis^a^		
							OR	95% CI	*p*	OR	95% CI	*p*
***Analysis 1 (N = 61 control and 51 intervention patients)***						
Adherence	47	(77.0)	46	(90.2)	13.1	(-5; 26.8)	2.74	0.91–8.23	0.065	3.68	1.14–11.88	**0.030**
Non-adherence	14	(33.0)	5	(9.8)								
***Analysis 2 (N = 80 control and 70 intervention patients)***						
Adherence	52	(65.0)	53	(75.7)	10.1	(-5.9; 26.0)	1.71	0.81–3.62	0.158	1.90	0.86–4.22	0.115
Non-adherence	27	(35.0)	17	(24.3)								

*Abbreviations: CI, confidence interval; OR, odds ratio. ^*a*^Using multivariable backward stepwise logistic regression models. Variables entered at the first step: intervention status, age, gender, health insurance, education grade ≥ 6, marital status, number of comorbidities ≥ 2, type of ACS, BMQ Specific Necessity and Concern scores, and PCI/CABG.*

There was a significant increase in median EQ-5D-3L index value from baseline to 3 months in both groups: control [0.686 (0546.; 1) vs. 1 (0.726; 1); *p* < 0.001] and intervention [0.766 (0.645; 1) vs. 1 (0.726; 1); *p* = 0.004]. But there was no significant difference in median change of EQ-5D-3L index values between control and intervention [0.234 (0.000; 0.379) vs. 0.000 (0.000; 0.275); *p* = 0.081] (**Table 4**).

**Table 4:** Changes in quality of life from baseline at the first 3 months after discharge.

	EQ-5D-3L	Baseline^a^				3-month^a^				Comparison^c^	
		Control^b^ *N* = 60, n (%)		Intervention^b^ *N* = 49, n (%)		Control^b^ *N* = 60, n (%)		Intervention^b^ *N* = 49, n (%)		*p_1_*	*p_2_*
Mobility	No problems	37	(61.7)	38	(77.6)	51	(85.0)	41	(83.7)	**0.004**	0.581
	Problems	23	(38.3)	11	(22.4)	9	(15.0)	8	(16.3)		
Self-care	No problems	50	(83.3)	43	(87.8)	55	(91.7)	47	(93.9)	0.227	0.289
	Problems	10	(16.7)	6	(12.2)	5	(8.3)	2	(4.1)		
Usual activities	No problems	46	(76.7)	41	(83.7)	55	(91.7)	46	(93.9)	**0.049**	0.180
	Problems	14	(23.3)	8	(16.3)	5	(8.3)	3	(6.1)		
Pain/discomfort	No problems	37	(61.7)	34	(69.4)	38	(63.3)	36	(73.5)	1.000	0.832
	Problems	23	(38.3)	15	(30.6)	22	(36.7)	13	(26.5)		
Anxiety/depression	No problems	35	(58.3)	30	(61.2)	45	(75.0)	36	(73.5)	0.052	0.238
	Problems	25	(41.7)	19	(38.8)	15	(25.0)	13	(26.5)		
EQ-5D-3L index value, median (IQR)		0.686	(0546.; 1)	0.766	(0.645; 1)	1	(0.726; 1)	1	(0.726; 1)	**<0.001**^d^	**0.004**^d^
Change of EQ-5D-3L index values, median (IQR)		*NA*		*NA*		0.234	(0.000; 0.379)	0.000	(0.000; 0.275)	*p_3_* = 0.081	

*Abbreviations: EQ-5D-3L, the European Quality of Life Questionnaire - 5 Dimensions - 3 Levels; IQR, interquartile range, NA, not applicable. *p*_*1*_ Comparison between baseline and 3-month EQ-5D-3L scores of the control group. *p*_*2*_ Comparison between baseline and 3-month EQ-5D-3L scores of the intervention group. *p*_*3*_ Comparison between changes from baseline of EQ-5D-3L index values of the control and intervention groups at the first 3 months after discharge. ^*a*^No difference in each dimension and index value of EQ-5D-3L between control and intervention groups at baseline or the first 3 months after discharge. ^*b*^Sample size of patients who reported EQ-5D-3L at both baseline and the first 3 months after discharge. ^*c*^Using McNemar test if other tests were not mentioned. ^*d*^Using Wilcoxon signed rank test.*

The proportion of patients dying, being readmitted to hospital, and both at 3 months was 0.8, 10.3, and 11.1%, respectively. These were not significantly different between the 2 groups (**Table 5**).

**Table 5:** Rates of mortality and readmission to hospital within the first 3 months after discharge.

			Group				
Adverse event	Overall *N* = 126, n (%)		Control *N* = 68, n (%)		Intervention *N* = 58, n (%)		*p-value^a^*
Mortality	1	(0.8)	1	(1.5)	0	(0)	1.000^b^
Readmission	13	(10.3)	6	(8.8)	7	(12.1)	0.551
Mortality/Readmission	14	(11.1)	7	(10.3)	7	(12.1)	0.752

*^*a*^Using Chi-square test if other tests were not mentioned. ^*b*^Using Fisher’s exact test.*

In **Analysis 2**, a greater proportion of patients were adherent in the intervention group compared with the control group at 1 month (adjusted OR = 2.35; 95% CI, 1.00–5.52), but no significant differences were found at 3 months (adjusted OR = 1.90; 95% CI, 0.86–4.22) (**Tables 2, 3**).

## Discussion

### Principal Findings

Our intervention comprising pharmacist-led medication counseling and tailoring, patient education, and providing medication aids enhanced the proportion of adherent patients by over 13% in the first 3 months after discharge. The potential factors associated with poor adherence were male gender and patients’ concerns about medicines. There was no statistically significant improvement in quality of life, mortality and readmission to hospital over the 3 months of the study.

### Strengths and Weaknesses of the Study

This is the first study to carry out an in-depth assessment of the pharmacist-led intervention that contributes to the improvement on adherence to cardioprotective medications in patients with ACS in Vietnam. A strength of our study was the high-quality design, e.g., allocation concealment to keep trial investigators unaware of upcoming allocations and blinded outcome assessment to avoid bias in estimated treatment effects. The MMAS-8 was translated, cross-culturally adapted and validated as a reliable tool to measure medication adherence in Vietnamese patients (Nguyen et al., 2015). In considering replication and expansion of this intervention in further large-scale studies or in clinical practice, there are several lessons about process and evaluation that may be useful. Our intervention included multiple components, all of which have been shown to improve adherence to medication regimens among patients with cardiovascular diseases (Cutrona et al., 2010; Ho et al., 2014). While our study was conducted in a specialized hospital, none of the components was unique to it and can be replicated in other health care settings. In our study, a pharmacist counseled prehospital and post-hospital medications within two weeks of discharge and provided patients with medication aids. The feasibility of this pharmacist-led intervention must be highlighted, as it does not depend on expensive materials or equipment to be performed. In fact, the role of pharmacists in Vietnam has been expanding from dispensing medications to providing services about medication management to support rational use of medicine (Vo et al., 2013). With an increasing number of patients needing the long-term use of secondary prevention medications for treatment of IHDs as well as other chronic diseases, findings of our study may encourage a closer cooperation of physicians with clinical pharmacists in order to make optimal use of available resources and achieve expected therapeutic outcomes in treatment for these patients.

Several issues in our study should be considered. **First**, we conducted the study in a selected single center setting in the urban region of Vietnam. Further research that includes more participants from multiple centers is needed to confirm the present results. Larger studies that include cost-effectiveness analyses are also needed. **Second**, the duration of the intervention might be too short to change a complex behavior such as adherence to taking medication. While we have seen changes in behavior in the expected direction in the intervention group of our study, we might have seen larger effects if the intervention had been repeated more often. Furthermore, studies with longer follow-up are needed to assess adherence on a long-term basis. **Third**, findings of the study might be difficult to compare to those of other studies due to the unique criteria for primary outcome measurement. **Fourth**, the study was powered to detect a difference of 20 percentage points in adherence. Although the pharmacist-led intervention had an effect on improved adherence, the effect size was smaller. This generated the wider confidence intervals of the estimated odds ratios. Larger studies are needed to confirm the efficacy and value of interventions with the small effect size. **Fifth**, we measured patient adherence using the MMAS-8 which might be biased by inaccurate patient recall or patient giving socially desirable responses. **Finally**, we asked patients about the adherence to all of their medication, evaluation of adherence to each individual medication is suggested for future studies.

### Possible Explanations and Comparison With Other Studies

A systematic review (Santo et al., 2016) showed that the delivery of an intervention, irrespective of its type, significantly improved medication adherence (OR = 1.52; 95% CI, 1.25–1.86). Many types of intervention components were implemented to improve medication adherence in patients with IHDs including patient education, counseling, intensified patient care, medication aids, simplification of drug regimen reminders, financial incentives, collaborative care, lay health mentoring, and direct observation treatment. In terms of modes of delivery, the interventions were delivered mostly by pharmacists, the others were nurses, researchers, or two health care professionals (Santo et al., 2016). Previous systematic reviews conducted to measure the effect of pharmacists on the care of patients with CVDs (Koshman et al., 2008; Santschi et al., 2011) and IHDs (Cai et al., 2013; Kang et al., 2016) have shown a positive impact on patient outcomes.

We found no significant effects on quality of life which is in line with previous studies (Community Pharmacy Medicines Management Project Evaluation Team, 2007; Calvert et al., 2012). Although a large proportion of all CVD events may be attributed to poor adherence to cardiovascular medications (Brown and Bussell, 2011; Chowdhury et al., 2013), we did not find a significant difference in mortality and readmission to hospital in our study, in line with some previous studies (Rinfret et al., 2013; Castellano et al., 2014; Ho et al., 2014; Khonsari et al., 2015). However, our study may have been too small and follow-up too short to observe these effects. In a study (Choudhry et al., 2011) the differences in clinical endpoints between full and usual prescription coverage began to diverge after 12 months.

## Conclusion

In conclusion, a pharmacist-led intervention improved adherence to cardioprotective medications in patients with ACS after hospital discharge, but not quality of life, mortality or readmission to hospital. These results are promising but should be tested in other settings prior to broader dissemination.

## Author Contributions

TN and KT wrote the first draft of the manuscript and are guarantors and take full responsibility for the integrity of the data and the accuracy of the data analysis. TN, KT, THN, PN, and HT participated in the design of the trial and study methodology. TN, PN, and HT performed the analysis. TN, THN, PN, HT, NN, HN, BH, TP, and KT reviewed the manuscript and made critical revisions. All authors read and approved the final manuscript.

## Conflict of Interest Statement

The authors declare that the research was conducted in the absence of any commercial or financial relationships that could be construed as a potential conflict of interest.
