# Supplementary material for: Pharmacist-Led Intervention to Enhance Medication Adherence in Patients With Acute Coronary Syndrome in Vietnam: A Randomized Controlled Trial
Source: Front Pharmacol. 2018 Jun 21;9:656. doi: 10.3389/fphar.2018.00656 (PMC6021484; doi:10.3389/fphar.2018.00656)
Supplement: Supplementary file 1 [file Data_Sheet_1.PDF]

Supplementary Material:

Pharmacist-led Intervention to Enhance Medication Adherence in Patients with Acute Coronary Syndrome in Vietnam: A Randomized Controlled Trial

Thang Nguyen*, Thao Huong Nguyen, Phu Thi Nguyen, Ha Thi Tran, Ngoc Viet Nguyen, Hoa Quoc Nguyen, Ban Ngoc Ha, Tam Thi Pham and Katja Taxis

***Correspondence:**
Dr. Thang Nguyen
nthang@ctump.edu.vn

1. Appendix 1. First counselling form and checklist

FIRST COUNSELLING FORM

**No**	**Pharmacist's Question/Answer**	**Patient's Answer**	**Possible explanation of pharmacist**
**Good morning (afternoon), my name is __________________. I'm a pharmacist of University of Medicine and Pharmacy at Ho Chi Minh city.<br>We met a few days ago, Mr./Ms. __________.<br>You look better now. Is it the good time to have discussion about your disease and the treatment?**			
**A1. ASSESSMENT OF DISEASE KNOWLEDGE**			
1	***What do you know about your disease?***		
2	***What do you know about the risk factors of your disease?***		
3	***What do you know about the possible cardiac events of your disease?***		
4	***What do you know about the prevention of your disease?***		
**A2. TAILORED ADVICE**			

**Definition**<br>Acute coronary syndrome is a term used for any condition brought on by sudden, reduced blood flow to the heart. Acute coronary syndrome symptoms may include the type of chest pressure that you feel during a heart attack, or pressure in your chest while you're at rest or doing light physical activity (unstable angina). The first sign of acute coronary syndrome can be sudden stopping of your heart (cardiac arrest). Acute coronary syndrome is often diagnosed in an emergency room or hospital.<br>**Risk factors**<br>1. Non-modifiable risk factors for atherosclerosis: increasing age, male, family history of premature coronary heart disease, premature menopause.<br>2. Modifiable risk factors for atherosclerosis: smoking, diabetes mellitus (and impaired glucose tolerance), hypertension, dyslipidemia (raised low-density lipoprotein (LDL) cholesterol, reduced high-density lipoprotein (HDL) cholesterol), obesity, and physical inactivity.<br>**Possible cardiac events**<br>Recurrent myocardial infarction, stroke, death related to any cardiovascular disease.<br>**Prevention**<br>1. Using your medications as physician's instructions on your prescription.<br>2. Frequently check your blood pressure, blood glucose and blood lipid.<br>3. Lifestyle modification: diet, physical exercise and smoking cessation.<br>**Teach back**<br>***I want to be sure I explained everything clearly. Can you please explain it back to me so I can be sure I did?***			
**B1. ASSESSMENT OF USING MEDICATIONS**			
5	***PAST EXPERIENCE***<br>***a) What medication did you take at home?***<br>***b) What type of difficulties have you encountered with regard to taking your medications in the past?***<br>***c) How did you deal with them?***		
6	***CURRENT CONCERNS OF TREATMENT***<br>***a) How do you feel about taking current medications?***		

	b) What are some reasons that you might have for taking the medications?<br>*c) What concerns you about taking them?*		
**B2. ENCOURAGEMENT AND TAILORED ADVICE**			
I strongly advise that it is important for your health that you take your medications each day as physician's instruction.<br>***The benefits of taking the medications in your case can prevent you from experiencing a heart attack.***<br>***Current treatment can help you:***<br>1. Relieve chest pain.<br>2. Prevent blood clots.<br>3. Prevent the buildup of and stabilize atherosclerotic plaque.<br>4. Restore blood flow through your heart.<br>**Teach back**<br>***Let's review what we discussed. What are four strategies that will help you control your ACS?***			
**C1. PROVIDE SUPPORT**			
I know it is sometimes hard to take the medications. I'd like to help you with this. I'm optimistic that you'll be able to take your medication and prevent the cardiac endpoints.<br>***Give instruction for using drug information leaflet.***<br>***Give instruction for using pill organizer***			
**C2. ADDRESS PROBLEMS/CONCERNS AND CORRECT MISUNDERSTANDING**			
7	***What concerns you about using these tools? Can you think of ways to deal with each of these problems you concern?***		
8	***Based on our discussion so far, what questions do you have?***<br>***We've gone over a lot of information. What are you going to do with these tools when you get home?***		
9	***I would like to call you to see how you are doing. What is the best time (day of week and time of day) to call you?***<br>***Schedule telephone calls and provide the patient with the list of dates and times the pharmacist will be calling.***<br>***Ask the patient to have all their prescriptions or medical records before them prior to the pharmacist call.***		
**<u>Note:</u>**			

CHECKLIST FOR THE FIRST COUNSELLING

Patient name:______________________________________ Study code: _______
Pharmacist name:____________________________________ Date: ____________
Start Time:________________________ End Time: _________________________

**No.**	**Counselling issue**	**Counsel**		**Teach back**	
		**Yes**	**No**	**Yes**	**No**
**A. ASSESSMENT OF DISEASE KNOWLEDGE AND TAILORED ADVICE**					
**1**	Assessment of disease knowledge				
**2**	Tailored advice on definition, risk factors, cardiac events and prevention of acute coronary syndrome				
**B. ASSESSMENT OF USING MEDICATIONS AND TAILORED ADVICE**					
**3**	Assessment of past experience of using medications for chronic disease				
**4**	Assessment of current concerns of treatment				
**5**	Encouragement and tailored advice on using medications				
**C. PROVIDE SUPPORT AND CORRECT MISUNDERSTANDING**					
**6**	Giving instructions for using drug information leaflet and pill organizer				
**7**	Address concerns and correct misunderstanding				
**8**	Schedule telephone calls				

Signature of Pharmacist

2. Appendix 2. Second counselling form and checklist

SECOND COUNSELLING FORM

**No**	**Pharmacist's Question/Answer**	**Patient's Answer**	**Possible explanation of pharmacist**
**Hello, this is Mr./Ms.______________ calling from the University of Medicine and Pharmacy at Ho Chi Minh city. Can I please talk to Mr./Ms. _____________ (state name of patient)? Mr./Ms.______________, we met about one week (two weeks) ago while you were in the hospital.**			
**A. ASSESSMENT OF GENERAL AND MEDICATION-RELATED ISSUES**			
1	***GENERAL ISSUES***<br>***a) How have you been doing?***		

	b) Is this a good time to talk about the treatment for your ACS?<br>***c) Do you have any questions about your disease?***<br>***d) Do you have all your medications and prescription in front of you?***		
2	***MEDICATION-RELATED ISSUES***<br>***a) How are you doing with your medications?***<br>***b) How do you take your medications?***<br>***c) What questions do you have about your medications?***<br>***d) The last time we talked, you mentioned that ____________ (name the problem the patient identified in the in-patient visit) had the potential to interfere with taking your medications. Was ________ a problem for you in the last (two) week(s)?***<br>***e) How did you deal with this (ask this question for each problem reported by the patient)? Have you experienced any other difficulty with regard to taking your medications?***<br>***f) How do you feel about taking your medications? (APA, BB, ACEI/ARB and statin).***<br>***g) It is helpful to identify your own reasons for doing something. What are some reasons that you might have for taking the medication?***<br>***h) Do you have any concerns about taking your medications? (APA, BB, ACEI/ARB and statin).***		
**B. ENCOURAGEMENT AND TAILORED ADVICE**			
I strongly advise that it is important for your health that you take the medications each day as prescribed by your health care provider.<br>***The benefits of taking the medications in your case can prevent you from experiencing a heart attack.***<br>***Tell patients the main role of their medications on their prescriptions.***<br>***Teach back: In your own words, please review what we talked about?***			
**C. PROVIDE SUPPORT AND CORRECT MISUNDERSTANDING**			

3	a) ***Have you used the leaflet and pill organizer?***<br>b) ***How are drug information leaflet and pill organizer helpful to you?***<br>c) ***What concerns you about using these tools?***<br>d) ***Did you think of ways to deal with each of these problems?***		
4	***Based on our discussion so far, what questions do you have?***		
I know it is sometimes hard to take your medications. I'd like to help you with this. I'm optimistic that you'll be able to take your medication and prevent the cardiac endpoints.			
**Note:**			

CHECKLIST FOR THE SECOND COUNSELLING

Patient name:____ Study code: _______
Pharmacist name:______________________________________ Date: _____________
Start Time:__________________________ End Time: ___________________________

**No.**	**Counselling issue**	**Counsel**		**Teach back**	
		**Yes**	**No**	**Yes**	**No**
**A. ASSESSMENT OF GENERAL AND MEDICATION-RELATED ISSUES**<br>**B. ENCOURAGEMENT AND TAILORED ADVICE**					
**1**	General issues				
**2**	Medication-related issues				
**3**	Encouragement				
**4**	Tailored advice on specific medications and/or problems				
**C. PROVIDE SUPPORT AND CORRECT MISUNDERSTANDING**					
**5**	Has been using drug information leaflet Yes ☐ No ☐				
**6**	Has been using pill organizer Yes ☐ No ☐				
**7**	Address concerns and correct misunderstanding				
**8**	Schedule telephone calls				

Signature of Pharmacist

3. Appendix 3. Process of data collection and management

No.	Step	Time	Description	Intervention	Control	Responsible investigator
1	Patient List Review	Hospitalization (after hospital admission)	Identified eligible patients from patient list of the hospital	All patients with an admission diagnosis of ACS		Investigators 1 or 2[a]
2	Recruitment	Hospitalization (patient at stable state)	Perfomed in-person interviews using data collection form 1, MMSE, informed consent	All patients having inclusion and no exclusion criteria		Investigators 3, 4 or 5[b]
3	Baseline data collection from patient interviews	Hospitalization (after recruitment)	Perfomed in-person interviews using BMQ-S, EQ-5D-3L	All patients having informed consent		Investigators 3, 4 or 5[b]
4	Randomizatio n	Hospitalization (after signing informed consent)	Performed randomization	All patients having informed consent were randomly allocated to control or intervention groups		Investigator 6
5	First counselling	Hospitalization	Performed the in-person counselling	Applied	NA	Investigators 3, 4 or 5[b]
6	Baseline data collection from medical records	Discharge	Collected data from medical records using data collection form 2	Applied	Applied	Investigators 3, 4 or 5[b]
7	Second counselling	Within 2 weeks after discharge	Performed the telephone counselling	Applied	NA	Investigators 3, 4 or 5[b]
8	Outcome measure 1	At 1 month after discharge	Perfomed a telephone interview using data collection form 3, BMQ-S, EQ-5D-3L, MMAS-8	Applied	Applied	Investigators 1 or 2[a]
9	Outcome measure 2	At 3 months after discharge	Perfomed a telephone interview using data collection form 3, BMQ-S, EQ-5D-3L, MMAS-8	Applied	Applied	Investigators 1 or 2[a]

Abbreviations: ACS, acute coronary syndrome; BMQ-S, the Beliefs about Medicines Questionnaire - Specific; EQ-5D-3L, the European Quality of Life Questionnaire - 5 Dimensions - 3 Levels ; MMAS-8, the 8-item Morisky Medication Adherence Scale; MMSE, the Mini–Mental State Examination; NA, not applicable

[a]Pharmacy students who assessed outcomes were blinded after assignment to intervention

[b]Pharmacists performed the intervention

4. Appendix 4. Description of nonadherence

Nonadherence	Overall N=126, n (%)		Group				
			Control N=68, n (%)		Intervention N=58, n (%)		*p-value*[a]
At one month after discharge							
Not complying with medical visits	18	(14.1)	13	(19.1)	5	(8.3)	0.080
MMAS-8 < 6	7	(5.5)	6	(8.8)	1	(1.7)	0.120[b]
At three months after discharge							
Complying with medical visits	17	(15.2)	12	(19.7)	5	(9.8)	0.147
MMAS-8 < 6	6	(5.4)	4	(6.7)	2	(3.9)	0.685[b]

Abbreviations: MMAS-8, the eight-item Morisky medication adherence scale
[a]Using Chi-square test if other tests were not mentioned
[b]Using Fisher's exact test
